# Long noncoding RNA expression signatures of bladder cancer revealed by microarray

**DOI:** 10.3892/ol.2014.1843

**Published:** 2014-01-31

**Authors:** YI-PING ZHU, XIAO-JIE BIAN, DING-WEI YE, XU-DONG YAO, SHI-LIN ZHANG, BO DAI, HAI-LIANG ZHANG, YI-JUN SHEN

**Affiliations:** 1 Department of Urology, Fudan University Shanghai Cancer Center, Fudan University, Shanghai 200032, P.R. China; 2 Department of Oncology, Shanghai Medical College, Fudan University, Shanghai 200032, P.R. China

**Keywords:** bladder cancer, long noncoding RNA, microarray

## Abstract

Dysregulation of long noncoding RNAs (lncRNAs) has been regarded as a primary feature of several human cancers. However, the genome-wide expression and functional significance of lncRNAs in bladder cancer remains unclear. The aim of this study was to identify aberrantly expressed lncRNAs that may play an important role in contributing to bladder cancer pathogenesis. In this study, we described lncRNAs profiles in four pairs of human bladder cancer and matched normal bladder tissues by microarray. We finally determined 3,324 differentially expressed human lncRNAs and 2,120 differentially expressed mRNAs (≥2-fold change). A total of 110 lncRNAs were significantly differentially expressed between the tumor and the control groups (≥8-fold change). Four lncRNAs (TNXA, CTA-134P22.2, CTC-276P9.1 and KRT19P3) were selected for further confirmation of microarray results using quantitative PCR (qPCR), and a strong correlation was identified between the qPCR results and microarray data. We also observed that numerous lncRNA expression levels were significantly correlated with the expression of tens of protein coding genes by construction of the lncRNA-mRNA co-expression network. Kyoto Encyclopedia of Genes and Genomes annotation showed a significant association with p53, bladder cancer, cell cycle and propanoate metabolism pathway gene expression in the bladder cancer group compared with the normal tissue group, indicating that deregulated lncRNAs may act by regulating protein-coding genes in these pathways. We demonstrated the expression profiles of human lncRNAs in bladder cancer by microarray. We identified a collection of aberrantly expressed lncRNAs in bladder cancer compared with matched normal tissue. It is likely that these deregulated lncRNAs play a key or partial role in the development and/or progression of bladder cancer.

## Introduction

Bladder cancer is the most common urinary tract malignant tumor and accounts for 5% of all diagnosed cancers (1). Although the majority of cases are not clinically advanced at presentation, the disease eventually recurs or develops metastases. Urothelial carcinoma of the bladder, the most common histopathological type of bladder cancer, has a variety of genetic and phenotypic characteristics. Numerous factors, including chromosomal anomalies, genetic polymorphisms, and genetic and epigenetic alterations, contribute to tumorigenesis and progression of urothelial carcinoma of the bladder (2). Therefore, revealing the molecular mechanism for bladder cancer development is required for developing effective therapy.

With the development of high-throughput DNA sequencing and array-based technologies, various classes of noncoding RNAs (ncRNAs) have recently been shown to function as regulators of protein-coding genes (3). Emerging data strongly suggest that long noncoding RNAs (lncRNAs; length >200 bp) are important in the basal regulation of protein coding genes, at the transcriptional and the posttranscriptional levels (3). Dysregulation of lncRNAs, such as HOTAIR, H19, MALAT-1 and PCA3, has been regarded as a primary feature of several human cancers, including prostate cancer, breast cancer, gastric cancer and hepatocellular carcinoma (4–7). Certain recent studies have reported that several lncRNAs, including UCA1, MALAT-1 and ncRAN, show marked potential in the field of bladder cancer progression (8–10). However, the genome-wide expression and functional significance of lncRNAs in bladder cancer remains unclear.

In this study, we present the lncRNA expression profiles in four pairs of bladder cancer samples and matched histologically normal urothelium samples by lncRNA microarray. We observed that a collection of lncRNAs were aberrantly expressed in bladder cancer compared with matched normal tissue, several of which were evaluated by quantitative PCR (qPCR) in a total of 51 pairs of tissues.

## Materials and methods

### Patient samples

Fifty-six patients with urothelial carcinoma of the bladder who received radical cystectomy at the Fudan University Shanghai Cancer Center (Shanghai, China) were included in the study. Urothelial carcinoma of the bladder was diagnosed histopathologically. Written informed consent was obtained from all patients and the study was approved by the Institutional Review Board of Fudan University Shanghai Cancer Center (Shanghai, China). Of the 56 pairs of samples, five pairs were used in lncRNA microarray analysis [one pair was excluded from analysis according to three dimension principal component analysis (3D-PCA)] and 51 pairs were analyzed by qPCR. The tumor sample and matched normal bladder tissue from each subject were snap-frozen in RNA ladder immediately after resection and stored in the tissue bank.

### RNA extraction

If the proportion of cancer cells in a tissue section was >80% then the frozen block was subjected to RNA extraction. Total RNA was extracted from 56 pairs of snap-frozen urothelial carcinoma and matched normal bladder tissues using TRIzol reagent (Invitrogen Life Technologies, Carlsbad, CA, USA) according to the manufacturer’s instructions. The RNA integrity was evaluated by a NanoDrop ND-1000 spectrophotometer (NanoDrop products, Wilmington, DE, USA).

### Microarray and computational analysis

RNA purified from total RNA following the removal of rRNA was amplified and transcribed into fluorescent cRNA along the entire length of the transcripts without 3′ bias utilizing a random priming method and cDNA was labeled and hybridized to the Human LncRNA Array V2.0 (8×60 K, Arraystar Inc., Rockville, MD, USA). A total of 33,045 lncRNAs and 30,215 coding transcripts, which were collected from the most authoritative databases, including RefSeq, UCSC Known Genes, Ensembl and the associated literature, were detected by the microarray.

The microarray work was performed by KangChen Bio-tech (Shanghai, China). In brief, the Arraystar lncRNA array protocol was as follows: i) the RNA sample, kit and reagents were prepared, including TRIzol reagent, Biopulverizer (Biospec, Bartlesville, OK, USA) and Mini-Bead-Beater-16 (Biospec); ii) Total RNA Clean-up and RNA quality control; iii) labeling reaction was prepared; iv) labeled/amplified RNA and labeled cRNA QC were purified; v) hybridization was performed; vi) microarray wash was conducted; vii) scanning was performed; and viii) the data were extracted using Agilent feature extraction software (Agilent Technologies, Santa Clara, CA, USA).

The arrays were scanned by the Agilent Scanner G2505B (Agilent Technologies) and the acquired array images were analyzed by Agilent Feature Extraction software (version 10.7.3.1; Agilent Technologies). Quantile normalization and subsequent data processing were performed using the GeneSpring GX v11.5.1 software package (Agilent Technologies).

### Gene ontology (GO) and pathway analysis

To discover the function and associated pathways of differentially expressed mRNAs, GO and pathway analyses were performed. GO annotations of microarray genes were downloaded from NCBI (http://www.ncbi.nlm.nih.gov/), UniProt (http://www.uniprot.org/) and the Gene Ontology (http://www.geneontology.org/). The elim Fisher algorithm was used to perform a GO enrichment test and GO categories with P<0.05 were reported. Pathway annotations of microarray genes were download from KEGG (http://www.genome.jp/kegg/) and a Fisher exact test was performed in order to locate the significant enrichment pathway. The resulting P values were adjusted using the Benjamini Hochberg false discovery rate (BH FDR) algorithm. Pathway categories with a FDR<0.05 were reported.

### Construction of the lncRNA-mRNA co-expression network

The network construction procedures included the following: i) preprocessing of data: if one coding gene has different transcripts the median value is taken to represent the gene expression values, without special treatment of lncRNA expression values; ii) data were screened and the subset of data were removed according to the lists of the differential expression of lncRNA and mRNA obtained from the GO and pathway analyses; iii) the Pearson correlation coefficient was calculated and the R value was used to calculate the correlation coefficient between lncRNA and coding genes; and iv) Pearson’s correlation coefficient was used for screening; RNAs with a Pearson’s correlation coefficient of ≥0.99 were considered significant and the lncRNA-mRNA co-expression network was constructed by Cytoscape software (The Cytoscape Consortium, San Diego, CA, USA).

### qPCR confirmation

To validate the microarray results, qPCR was performed. Total RNA was extracted from frozen tumor specimens using TRIzol reagent and then reverse transcribed using the Maxima Probe qPCR Master mix (Fermentas, Waltham, MA, USA) according to the manufacturer’s instructions. lncRNA expression levels in bladder cancer tissues were measured by qPCR using a GeneAmp PCR System 9700 (Applied Biosystems, Waltham, MA, USA). The primers used in this study are shown in Table I. Four lncRNAs that were significantly deregulated (TNXA, CTA-134P22.2, CTC-276P9.1 and KRT19P3) were evaluated in the patients included in this study.

Total RNA (2 μg) was converted to cDNA according to the manufacturer’s instructions. PCR was performed in a total reaction volume of 20 μl, including 10 μl Master Premix (2X, with ROX Reference Dye II), 1 μl of PCR Forward Primer (10 μM), 1 μl of PCR Reverse Primer (10 μM), 0.2 μl Roche probe (100X), 2 μl of cDNA and 5.8 μl of double-distilled water. The qPCR reaction was set at an initial denaturation step of 10 min at 95°C; and 95°C (15 sec), 57°C (30 sec), 72°C (30 sec) for a total 40 cycles. The experiments were performed in triplicate. The samples were normalized to β-actin. The median in each triplicate was used to calculate relative lncRNA concentrations (ΔCt = Ct median lncRNAs - Ct median β-actin). Expression fold changes were calculated using the 2^−ΔΔCt^ method.

### Statistical analysis

Statistical analysis was performed using Student’s t-test for the comparison of the two groups in microarray, and analysis of variance for multiple comparisons. P<0.05 was considered to indicate a statistically significant result. The statistical significance of the microarray result was analyzed by fold change and Student’s t-test. The FDR was calculated to correct the P-value. The threshold value used to screen differentially expressed mRNAs was a fold change of ≥2.0, and a fold change of ≥8.0 for differentially expressed lncRNAs.

## Results

### Overview of lncRNA profiles

The expression profiles of lncRNAs in paired samples were shown by calculating the log_2_ fold-change tumor/normal (T/N). The agreement was formulated as follows: Fold change (FC) cut-off: 2.0. Positive fold change values indicated upregulation and negative values indicated downregulation. Log fold change means log_2_ of absolute fold-change (log_2_FC). The fold change and P-value were calculated from the normalized expression. One pair of samples was excluded from analysis according to 3D-PCA. We finally identified 3,324 differentially expressed human lncRNAs in four bladder cancer patients (≥2-fold).

A total of 110 lncRNAs were significantly differentially expressed between the tumor and control groups (≥8-fold). Twenty-two lncRNAs were upregulated and 88 lncRNAs were downregulated in the tumor group compared with the controls (Table II). Log_2_FC of upregulated lncRNAs in the tumor group ranged between 3.017291 to 4.581319, and −3.00191 to −6.10723 of downregulated lncRNAs. RP11-58A12.3 (log_2_FC=−6.10723) was the most significantly downregulated lncRNA and RNU12 (log_2_FC=4.581319) was the most significantly upregulated lncRNA. We observed that downregulated lncRNAs were more common than upregulated lncRNAs.

### Overview of mRNA profiles

**Figure 1:**
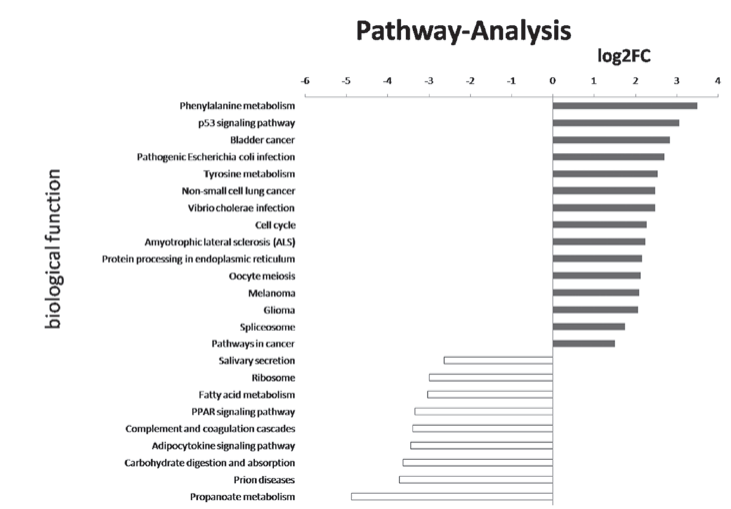
Pathway analysis. Kyoto Encyclopedia of Genes and Genomes annotation showed a significant association with p53, bladder cancer, cell cycle and propanoate metabolism pathway gene expression in the bladder cancer group compared with normal tissue group. Log_2_ fold change (log_2_FC), a positive value indicates upregulation and negative value indicates downregulation.

Up to 17,069 coding transcripts were detected in four pairs of samples using 30,215 coding transcripts probes. Among the four pairs of samples, 1,269 mRNAs were upregulated in tumor samples compared with the matched normal tissues, while 851 mRNAs were downregulated. Kyoto Encyclopedia of Genes and Genomes (KEGG) pathway analysis showed that the differentially expressed mRNAs may be associated with p53, bladder cancer, cell cycle and propanoate metabolism pathways (Fig. 1). These results suggest that bladder cancer has a variety of genetic and phenotypic characteristics.

### Confirmation of microarray results by qPCR

**Figure 2:**
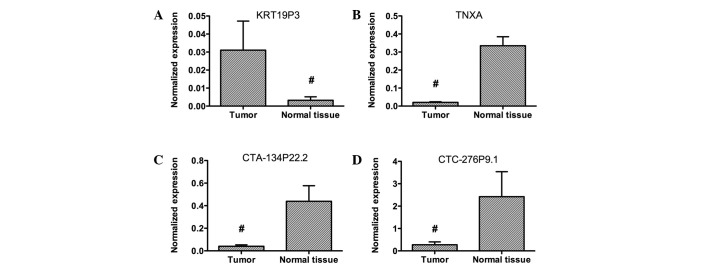
Confirmation of (A) KRT19P3, (B) TNXA, (C) CTA-134P22.2 and (D) CTC-276P9.1 lncRNA levels by quantitative PCR (qPCR). qPCR analysis confirmed microarray data. After normalization to β-actin RNA, data were presented as mean ± SD (n=51) and the median value for each lncRNA was used for statistical analysis. The experiment was conducted in triplicate. ^#^P<0.05 versus control. lncRNA, long noncoding RNA.

**Figure 3:**
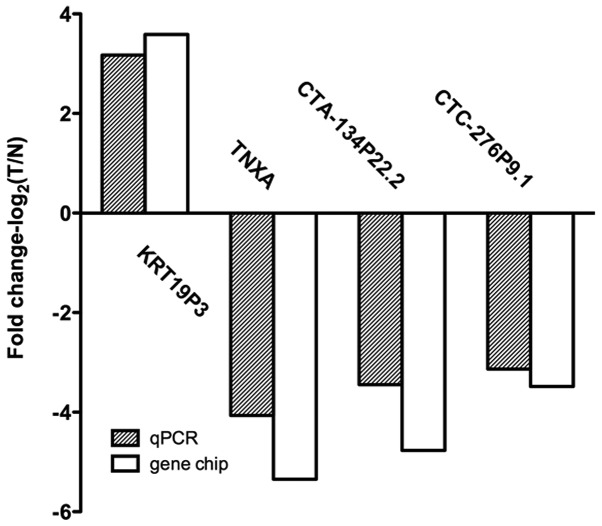
Comparison between microarray data and quantitative PCR q(PCR) result. The heights of the columns in the chart represent the log-transformed median fold changes (T/N) in expression across the four patients for each of the four confirmed lncRNAs. The confirmed results of the four lncRNAs indicated that the microarray data correlated well with the qPCR results. T/N, tumor/normal.

Four lncRNAs were selected for further confirmation of microarray results using qPCR. These lncRNAs were among the most downregulated or upregulated lncRNAs. Data analysis showed that KRT19P3 was upregulated and TNXA, CTA-134P22.2 and CTC-276P9.1 were downregulated in bladder cancer samples compared with matched normal tissues (Fig. 2). These data support a strong correlation between the qPCR result and the microarray data (Fig. 3).

### Construction of the lncRNA-mRNA co-expression network

We constructed a coding-non-coding gene co-expression network based on the correlation analysis between the differentially expressed lncRNA and mRNA. We used lncRNAs and mRNAs with Pearson’s correlation coefficients of no less than 0.99 to construct the network. In total, 79 lncRNAs and 103 mRNAs were included in the co-expression network. The co-expression network indicated that one mRNA or lncRNA may correlate with one to tens of lncRNAs. The co-expression network may suggest that the inter-regulation of lncRNAs and mRNAs is involved in bladder cancer.

## Discussion

The human transcriptome is more complex than a collection of protein-coding genes and their splice variants (11–13). With the advent of whole-genome and transcriptome sequencing technologies, it was shown that at least 90% of the genome is actively transcribed (13). Although lncRNA was initially suggested to be transcriptional noise, recent evidence suggests that this part of the genome may play a major biological role in cellular development and human diseases (14,15). The newly discovered lncRNAs demonstrate developmental and tissue-specific expression patterns, and aberrant regulation in a variety of diseases, including cancer (16–18). However, the function of lncRNAs in tumor pathogenesis and growth is less well characterized.

Recent studies have started to reveal the importance of lncRNAs in tumorigenesis in bladder cancer. Yang *et al* demonstrated that UCA1 regulates cell cycle progression through CREB via PI3K-AKT-dependent signaling pathways and may serve as a new diagnostic and therapeutic target in bladder cancer (8). Ying *et al* demonstrated that MALAT-1 expression levels were upregulated in bladder cancer that subsequently metastasized, and that increased expression of MALAT-1 activated the Wnt pathway to promote epithelial-mesenchymal transition and human bladder cancer cell metastasis (9). However, the genome-wide expression and functional significance of lncRNAs in bladder cancer remains unclear.

In this study, we evaluated the lncRNA expression profile in the tissue of four bladder cancer patients to reveal the potential role of lncRNAs in the pathogenesis of bladder cancer. Microarray techniques revealed a set of significantly differentially expressed lncRNAs, with 22 upregulated and 88 downregulated lncRNAs in bladder cancer tissue compared with matched normal tissue (fold change ≥8). For further validation of microarray results, qPCR was performed to evaluate the expression patterns of TNXA, CTA-134P22.2, CTC-276P9.1 and KRT19P3 in a total of 51 patients with bladder cancer. The qPCR results matched well with the data from the microarray.

A major function of lncRNAs is to modulate the epigenetic status of proximal or distal protein-coding genes via cis- and trans-acting mechanisms (19,20). We also observed that numerous lncRNA expression levels were significantly correlated with the expression of tens of protein coding genes by construction of the lncRNA-mRNA co-expression network. For example, RUNX1T1 and SLC25A4 were correlated with 21 and 28 mRNAs respectively.

In order to gain insight into the function of targets of lncRNAs, GO term and KEGG pathway annotation were applied to the target gene pool. KEGG annotation showed a significant association with the p53, bladder cancer, cell cycle and propanoate metabolism pathway gene expression in the bladder cancer group compared with the normal tissue group, indicating that deregulated lncRNAs may act by regulating protein-coding genes in these pathways.

We demonstrated for the first time the expression profiles of human lncRNAs in bladder cancer by microarray. We identified a collection of aberrantly expressed lncRNAs in bladder cancer compared to matched normal tissue. It is likely that these deregulated lncRNAs play a key or partial role in the development and/or progression of bladder cancer. Further study is required to determine whether these lncRNAs may serve as new therapeutic targets and diagnostic biomarkers in bladder cancer.

## Figures and Tables

**Table I:** Oligonucleotide primer sequences.

	Quantitative PCR primer (5′ to 3′)		
		
Primer set name	Forward	Reverse	Probe no. (Roche)
TNXA	acgtgttttgggacatgga	caaaaccatgggcatagtcc	20
CTA-134P22.2	ggggatggaagatggtgtc	aagggtgggctctcatctg	49
CTC-276P9.1	ccgaaacctgagccagag	cctctctcctgcccacttc	44
KRT19P3	agctcgccacctacctcag	ggaggtggacaggctattgt	72

**Table II:** Deregulated lncRNAs detected using microarray in four bladder cancer patients.

Downregulated in cancer		Upregulated in cancer	
	
lncRNAs	Log_2_ fold change (T/N)	lncRNAs	Log_2_ fold change (T/N)
RP11-58A12.3	−6.10723	RNU12	4.58132
LOC572558	−5.95465	KRT42P	4.56141
TNXA	−5.34750	COTL1P1	4.23520
LOC100302650	−5.26266	lincRNA-RCN2	4.11605
ADCY5	−5.24113	RP11-263F15.1	3.72144
DCLK1	−5.07589	LOC400879	3.70993
RP11-14D22.5	−4.97210	KRT19P3	3.58818
ADCYAP1R1	−4.92745	DUXAP10	3.54649
CTA-134P22.2	−4.76654	uc.30	3.48551
AB074188	−4.49578	keratin 19	3.47475
AL390170	−4.34162	RP5-1100H13.3	3.22775
ADAM22	−4.20736	GATA3	3.17360
CR621436	−4.06665	lincRNA-ZNF672	3.16483
AP1S2	−4.06335	KRT8P10	3.14700
LPHN3	−4.04289	RP11-133K18.1	3.13581
LOC284276	−4.03413	RP11-184B22.2	3.07165
XIST	−3.96822	KRT8P25	3.04478
LOC400550	−3.95690	KRT8P18	3.03897
CR605298	−3.91532	HMGA1P2	3.02231
C10orf108	−3.88202	KRT16P1	3.01729

This table only lists the 20 most upregulated and downregulated lncRNAs. lncRNA, long noncoding RNA; T/N, tumor/normal.
